# Real-time monitoring of CAR T cell dynamics in tumor patient-derived organoids using the OrganoIDNet algorithm

**DOI:** 10.1186/s12967-026-08929-x

**Published:** 2026-09-09

**Authors:** Nathalia Ferreira, Camille Dourlens, Riccardo Scodellaro, Philipp Stroebel, Daniel Schäfer, Olaf Hardt, Frauke Alves

**Affiliations:** 1 Translational Molecular Imaging, Max-Planck-Institute for Multidisciplinary Sciences, 37075 Göttingen, Germany; 2 Miltenyi Biotec B.V. & Co. KG, 51429 Bergisch Gladbach, Germany; 3 Clinic of Hematology and Medical Oncology, University Medical Center Göttingen, 37075 Göttingen, Germany; 4 Institute of Pathology, University Medical Center Göttingen, 37075 Göttingen, Germany; 5 Institute for Clinical and Interventional Radiology, University Medical Center Göttingen, 37075 Göttingen, Germany

**Keywords:** Patient-derived organoids, CAR T cells, PDAC, Artificial intelligence, OrganoIDNet algorithm, CD318

## Abstract

**Background:**

Patient-derived organoids (PDOs) provide physiologically relevant 3D tumor models for preclinical drug testing, yet robust and automated methods to quantify dynamic responses to immunotherapies remain limited. OrganoIDNet is a deep learning-based image analysis framework that enables automated, label-free segmentation and longitudinal quantification of organoid morphology. Here, we extend the application of OrganoIDNet to evaluate chimeric antigen receptor (CAR) T cell activity against pancreatic ductal adenocarcinoma (PDAC) PDOs targeting the tumor-associated antigen CD318.

**Results:**

CD318-directed CAR T cells were co-cultured with PDAC PDOs using a Matrigel-based sandwich system and monitored by time-lapse bright-field imaging. OrganoIDNet enabled accurate single-organoid segmentation and continuous quantification of organoid number and area across multiple effector-to-target ratios. CAR-318 T cells induced robust, antigen-dependent cytotoxicity, characterized by progressive reductions in organoid number and size and were accompanied by increased T cell activation marker expression and changes in TIM-3 expression at the endpoint. Dynamic imaging and T cell spatial analysis, further revealed close T cell-organoid interactions and early tumor cell elimination, providing time-resolved information on organoid responses and T cell proximity that complements conventional endpoint assays.

**Conclusions:**

By integrating organoid-immune co-cultures with OrganoIDNet-driven live-cell imaging, we established an automated longitudinal imaging assay for assessing CAR T cell-mediated responses in PDAC PDOs. The assay enabled continuous quantification of organoid number and area, together with image-based assessment of T cell proximity, across multiple effector-to-target ratios. These measurements provide time-resolved information on antigen-dependent cytotoxicity and spatial association during the observation period, supporting its potential utility as a preclinical platform for CAR T cell development and future personalized immunotherapy studies.

**Supplementary Information:**

The online version contains supplementary material available at https://doi.org/10.1186/s12967-026-08929-x.

## Background

Patient-derived organoids (PDOs) represent an attractive model for cancer research, offering accurate in vitro recapitulation of the genetic and physiological features of patient tumors [1]. This 3D in vitro system has shown robust utility across various tumor types, including pancreatic ductal adenocarcinoma (PDAC) [2], a particularly aggressive malignancy with limited therapeutic options [3]. While chimeric antigen receptor (CAR) T cell therapy has been highly effective in hematological cancers, its application to solid tumors like PDAC is hindered by the immunosuppressive microenvironment and tumor antigen heterogeneity [4]. Recently, a comprehensive screen of tumor-specific antigens suitable for CAR T cell therapy in PDAC identified CD318 as a promising candidate. CAR-318 T cells demonstrated potent in vivo tumor-killing effects and exhibited a favorable safety profile, supporting the potential of CD318 as a target for clinical translation [5].

Given the use of PDOs as rapid, physiologically relevant preclinical models, we assessed CAR T efficacy by employing our established mixed cell culture system, which involves human PDAC PDOs co-cultured with or without immune cells. In combination with OrganoIDNet, our previously developed image-analysis algorithm, we evaluated whether automated segmentation and longitudinal bright-field imaging could provide quantitative measurements of organoid morphology and T cell proximity during short-term CAR T cell co-culture.

## Materials and methods

### Cell lines, culture conditions and Knock Out (KO) generation

AsPC1 (CRL-1682), BxPC3 (CRL-1687) and MCF-7 (ACC 115) cells were obtained from ATCC and cultured as recommended. These cells were cultivated in RPMI media (Biowest, L0501), supplemented with 2mM L-glutamine from Gibco and 10% Fetal Calf Serum (FCS) (Eximus, BS-2020-500). For co-culture with T cells, AsPC1 and BxPC3 cells were transduced to express firefly luciferase (Luc) and green fluorescent protein (GFP). The AsPC1 KO CD318, AsPC1 KO TSPAN8 and BxPC3 KO CD318 cell line variants were generated by CRISPR-Cas9 followed by electroporation using the CliniMACS^®^ Electroporator integrated to the CliniMACS Prodigy^®^ instrument (Miltenyi Biotec). All sgRNAs were pre-designed and ordered from Integrated DNA Technologies (IDT), as well as the Cas9 Nuclease V3 and electroporation enhancer, using manufacturer recommendation. Human embryonic kidney 293T cells (HEK293T, ACC 635) were obtained from the DSMZ-German Collection of Microorganisms and Cell Cultures and cultivated in DMEM High Glucose media (Biowest, L0104), supplemented with 10% FCS.

### Generation of CAR plasmids

CD318 CAR and TSPAN8 CAR were cloned in silico using the Geneious Prime software (version 2023.0.1). They all comprised murine scFvs specific for CD318 or TSPAN8, that were preceded by a CD8α leader peptide. Heavy and light chains of the scFvs were connected with a glycine-serin linker. The binding domains were followed by spacers differing in size (XS or S). The library of backbones comprised either a human IgG4 hinge from the sequence between CH2 and CH1 domain (XS; extra short spacer; 12 aa), or a human CD8α spacer (S; short spacer; 45 aa). All CARs shared the same CD8α transmembrane domain and featured 4-1BB/CD3ζ-derived intracellular signaling domains. The CAR sequence was linked to a P2A sequence leading to co-expression of truncated LNGFR.

### Lentiviral particle production

VSV-G pseudotype lentiviral particles encoding CAR sequences were generated by transfection of HEK293T cells using pMDG2 (encoding VSV-G), pCMVdR8.74 (encoding gag/pol), and the respective transgene-encoding transfer vector. 24 h after transfection, 10 mM Sodium Butyrate (Sigma Aldrich, CAS N°: 156-54-7) was added to the culture medium. Lentiviral particles were harvested 48 h after transfection by passing the cell culture supernatant through a 0.45 μm filter and centrifugation over night at 4 °C. Lentiviral particles were resuspended in TexMACS™ Medium (Miltenyi Biotec, 170-076-306) and stored at -70 °C until transduction.

### Isolation of T cells and generation of CAR T cells

Peripheral blood mononuclear cells (PBMCs) were isolated by PanColl (PanBiotech, P04-66500) density gradient centrifugation from the whole blood of healthy anonymous donors. T cells were purified from PBMCs using the Pan T Cell Isolation Kit, human (Miltenyi Biotec, 130-096-535) and activated in TexMACS™ Medium (Miltenyi Biotec, 170-076-306) containing T Cell Trans-Act™, human (Miltenyi Biotec, 130-111-160) and 12.5 ng/mL of both recombinant human interleukins IL-7 and IL-15 (Miltenyi Biotec, respectively 130-095-367 and 130-095-760). T cells were transduced 24 h after activation using lentiviral particles. Three days post activation, T Cell TransAct™, human, was washed out of the medium, and T cells were cultured with IL-7 and IL-15 containing TexMACS™ Medium. Approximately 10 days after purification from PBMCs, T cells were frozen with TexMACS™ Medium supplemented with 10% of Fetal Calf Serum (FCS, Eximus^®^, BS-2020-500) and 10% of Dimethylsulfoxid (DMSO, Sigma-Aldrich^®^, D8418) until further use for in vitro testing. Frozen T cells were thawed 24 h before use in TexMACS™ Medium with IL-7 and IL-15. On the day of use, the number of viable CAR T cells was determined by flow cytometry following staining with 7-AAD and anti-human low-affinity nerve growth factor receptor (LNGFR) (Miltenyi Biotec, 130-111-568 and 130-112-602, respectively).

### T cell co-culture assays with tumor cell lines

Target cells were inoculated in 96- well culture plates at densities of 1.5 or 2 × 10^4^ cells per well in 100 µL of TexMACS™ Medium without cytokines (Miltenyi Biotec, 170-076-306). Directly thereafter, CAR^+^ T cells were added in various E: T ratios. Final vessel volume equaled 200 µL. The amount of T cells in the non-transduced control (NTC) was adjusted to the number of total T cells in the CAR group with the highest total cell count. Cytotoxicity was measured as a decrease in green surface area with the Incucyte^®^ S3 Live-Cell Analysis System (Sartorius) and the supplied software Incucyte S3 (versions 2019 A and 2023 A Rev2). The green surface area was normalized to 1 based on the first measurement and after each rechallenge. The following decrease or increase in surface area was set into relation.

### PDAC organoid establishment and culture

Human PDAC patient-derived organoids (PDOs) were generated from resected pancreatic cancer tissue from seven different patients as previously described [6]. Organoids were cultured in PDAC organoid medium (Stem Cell Technologies, 100–0781) embedded in Matrigel domes. As part of the Clinical Research Unit 5002 (CRU5002), the PDOs used in this study are listed in Supplementary Table S1.

### PDAC organoid-T cell co-culture assays

Co-cultures of human PDAC PDOs and CAR T cells were made following the protocol described before [7]. Briefly, organoids from six confluent wells were enzymatically dissociated using TrypLE-EDTA (Sigma-Aldrich, T3924), counted, and replated with 10,000 cells in 20 µL Matrigel (Corning, 356231) domes. After three days of organoid maturation, and on the day of co-culture, PDAC PDOs were gently removed from the Matrigel by pipetting the organoid suspension up and down using a 10 mL pipette fitted with a yellow end-cut tip. Co-cultures were established at effector-to-target (E: T) ratios of 2:1, 5:1, and 8:1, where E: T denotes the ratio of CAR T cells to tumor cells. For the co-culture setup, 30 µL of 25% Matrigel (in DMEM (Gibco, 41966-029)) was added to 96-well plates and solidified. T cell-organoid mixtures were added (20 µL), overlaid with an additional Matrigel layer, and topped with 200 µL of 1:1 PDAC organoid: TexMACS™ Medium (without cytokines).

### Live-imaging of PDAC organoid-T cell co-cultures

Organoid growth and T cell-mediated cytotoxicity were monitored using the Incucyte^®^ S3 Live-Cell Analysis System (Sartorius). Brightfield images of each well in the 96-well plate were captured every 4 to 48h using a 4X objective and the organoid imaging mode described before [8], enabling continuous tracking of Matrigel-embedded organoids and providing real-time, label-free measurements of organoid growth and responses.

### OrganoIDNet analysis

PDAC organoid-T cell co-culture images were analyzed using OrganoIDNet, a previously published deep-learning model for organoid segmentation and quantification in organoid–immune-cell co-cultures [8]. In the present study, the pretrained model was used in inference mode and was not retrained or revalidated. The model architecture, training dataset, data partitioning, quantitative performance evaluation, comparison with alternative segmentation approaches, and robustness analyses are described in detail in [9]. Briefly, OrganoIDNet is based on a fine-tuned StarDist architecture with a U-Net backbone and additional convolutional layers. In the present analysis, the model was used to quantify organoid number and area at each imaging time point. Organoids that were not captured by the segmentation mask or were only partially imaged were excluded. Measurements were normalized to the corresponding initial value to account for differences between wells and starting organoid number or area.

### Code and Data availability

The codes used in this study are publicly available in dedicated GitHub repositories (https://github.com/ajinkya-kulkarni/PyOrganoIDNet and https://github.com/ajinkya-kulkarni/PyBlenPatches) under the CC-BY 4.0 license. These repositories contain the implementation of the OrganoIDNet framework and associated tools. In addition, the pre-trained model weights and the dataset used for training and evaluation are freely accessible through the corresponding Zenodo repository [10].

### T cells segmentation and proximity analysis

We segmented T cells by using a pipeline for small objects detection. Images were first processed with a band-pass filter implemented as the difference between two Gaussian blurs (σ = 1 and 7 pixels) to improve background uniformity. We binarized the filtered images with Otsu thresholding, and we identified the connected components. Objects with a small area, between 3 and 40 pixels, were considered as T cells. Based in the previously published proximity analysis [11], we quantified T cell proximity to organoids by generating a defined neighborhood region around each organoid segmented using OrganoIDNet. We defined this region as the set of pixels located outside the organoid boundary within a distance of 70 pixels, computed using a Euclidean distance transform. Then, we quantified the number of segmented T cells intersecting this neighborhood region. To assess whether the observed T cell accumulation exceeded random spatial expectation, we implemented a null model by randomly relocating the neighborhood region within the image 5 times while preserving its geometry. For each image, we averaged the observed number of T cells and the corresponding null-model results across all detected organoids. We computed mean values and standard errors of the mean. Finally, we aggregated these measurements across samples and time points to obtain the final quantitative metrics.

### Confocal microscopy

Live cell imaging was performed using a Zeiss LSM880 confocal microscope. T cells were pre-labeled 4 h before the co-culture with BioTracker Cystine-FITC (Sigma, 5 µM, SCT047). The fluorescent-labeled antibody against EpCAM-AF594 (Biolegend, 1:100; 324228) was added in a post-co-culture setup for tumor organoid staining. Imaging was conducted at 37 °C and 5% CO₂ using 488 and 594 nm laser lines for FITC and AF594 excitation, respectively. Data analysis was performed using Imaris (version 10).

### Flow cytometry

Samples from the PDAC cells, PDAC PDOs alone or PDAC PDOs/CAR T cells were frozen with a 90% FCS and 10% DMSO solution and stored at -80 °C until the flow cytometry characterization. Single-cell suspensions were stained with antibody conjugates and blocked with human FcR reagent from Miltenyi Biotec, as recommended by the supplier. Antibody details are listed in Table 1. Therefore, conjugates were added to cell suspensions in a final ratio of 1:100, 1:11 or 1:50 and incubated 10 min at 4 °C. The staining buffer used was PEB, prepared from autoMACS^®^ Rinsing solution (Miltenyi Biotec, 130-091-222) containing PBS and 2 mM EDTA, supplemented with 0.5% MACS^®^ BSA Stock Solution (Miltenyi Biotec, 130-091-376). After the incubation, a washing step was performed adding PEB in an excess of minimal 5 times the staining solution. Cells were spun down and resuspended in appropriate volumes of PEB and measured on a flow cytometer. Dead cells were excluded using 7-AAD (Miltenyi Biotec, 130-111-568). AsPC1 cells, BxPC3 cells and PDOs were characterized by the expression of CD318 and TSPAN8. Samples were analyzed using a BD LSR Fortessa-20 cytometer (Becton Dickinson) and MACSQuant^®^ Analyzer 16 Flow Cytometer. Data analysis was performed using FlowJo (version 10.10.0) and MACSquantify (version 2.13.0). Gating strategies for CD318 and TSPAN8 expression in PDAC cells or PDOs are shown in Supplementary Fig. S2. Gating strategy for organoid and CAR T cell phenotyping after co-cultures is shown in Supplementary Fig.S3.

**Table 1:** List of antibodies used for flow cytometric analysis

Target	Conjugate	Catalog number
FcR blocking reagent	-	130-059-901
CD326 (EpCAM)	FITC	130-110-998
CD271 (LNFGR)	APC	130-110-998
CD366 (TIM-3)	PE-Vio770	130-121-334
CD69	APC-Vio770	130-112-616
7-AAD	-	130-111-568
CD318	PE	130-101-215
CD318	APC	130-101-249
TSPAN8	PE	130-117-391

### Statistical analysis

Statistical analyses were performed using GraphPad Prism 10.2.3. The experimental unit was the independent biological replicate, defined by the CAR T cell donor, independent experiment, or independent PDO preparation, as specified in the corresponding figure legend. Technical replicate wells were averaged before statistical testing and were not treated as independent biological replicates.

For comparisons involving more than two groups with a common control, one-way ANOVA followed by Dunnett’s test for multiple comparisons was used. Dunnett’s procedure was selected because the primary comparisons were treatment conditions versus a prespecified control, including untreated, non-transduced T cell, wild-type, or antigen-knockout controls, as appropriate. For comparisons between two independent groups, two-sided unpaired Student’s t-tests were used. Where multiple conditions or endpoints were tested, multiplicity-adjusted P values from the corresponding post hoc procedure are reported. Statistical tests and the relevant control group are specified in each figure legend.

Data are presented as mean ± SD unless otherwise indicated. Statistical significance was defined as (*P* < 0.05). The number of independent biological replicates is provided in the figure legends.

## Results

**Fig. 1:**
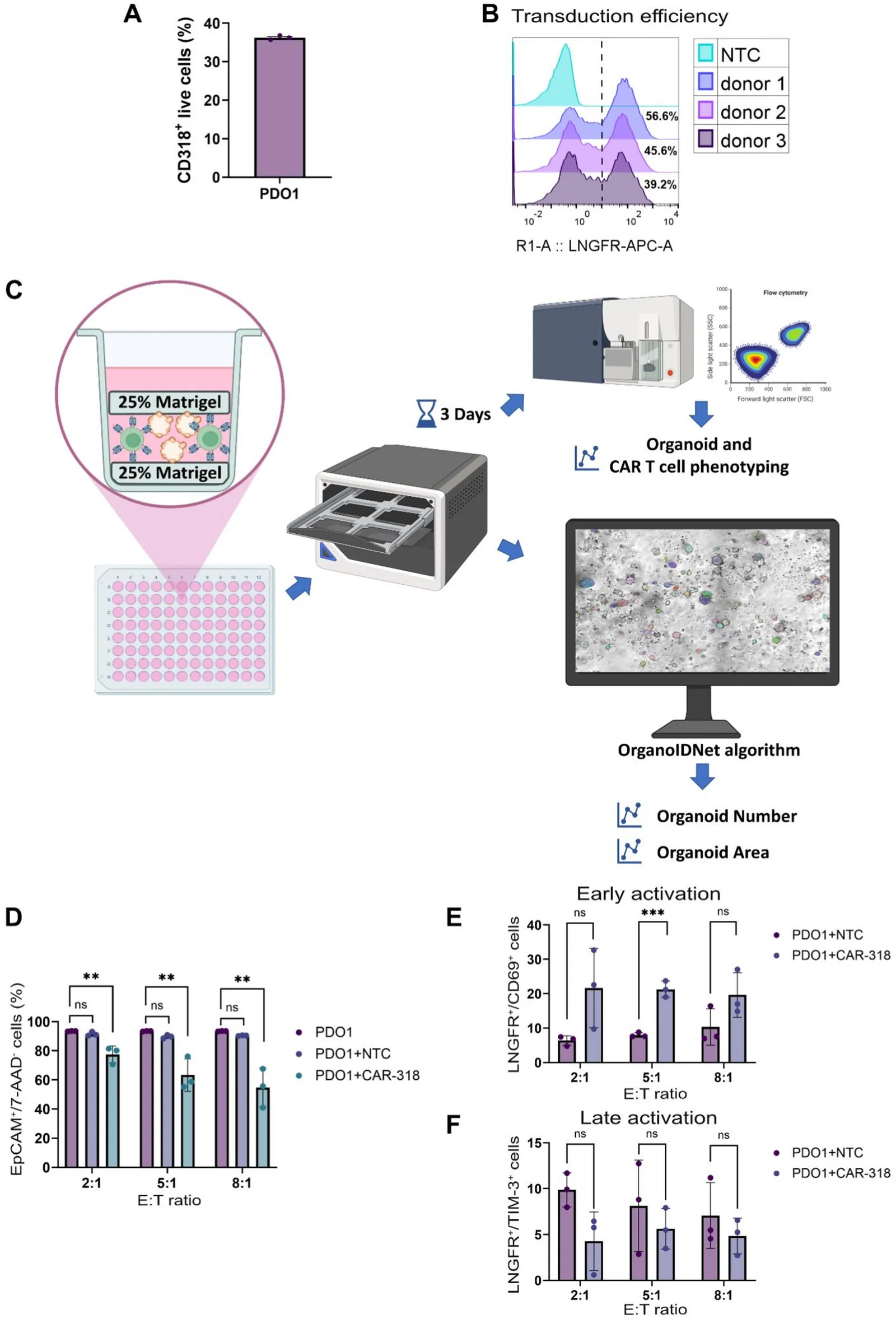
Flow cytometric analysis of tumor and immune cell populations in human PDAC PDO organoid co-cultures with CAR-318 T cells after 3 days. **(A)** Quantification of live CD318^+^ tumor cells as a percentage of total live tumor cells in PDO1 PDAC organoids. Data represent mean ± S.D. from two independent experiments. **(B)** Histogram showing CAR-318 transduction efficiency in T cells from three healthy donors (donors 1–3), assessed by LNGFR expression. Non-transduced T cells (NTCs) serve as controls. **(C)** Schematic workflow for the in vitro evaluation of CAR T cell dynamics during co-culture with PDAC PDOs. OrganoIDNet enabled automated segmentation and quantification of organoid responses to CAR T cells. At the end of the co-culture period, flow cytometry was performed to characterize both PDO populations and CAR T cell activation states. **(D)** Frequency of EpCAM⁺/7-AAD^−^ tumor cells as a percentage of total live tumor cells in PDO1 after 3-day co-culture with CAR-318 T cells (PDO1 + CAR-318), non-transduced control T cells (PDO1 + NTC), or in the absence of T cells (PDO1) at varying effector-to-target (E: T). **(E)** Proportion of LNGFR⁺/CD69⁺ double-positive cells indicating early T cell activation following 3-day co-culture of CAR-318 or NTC T cells with PDO1 PDAC organoids at different E: T ratios. **(F)** Proportion of LNGFR⁺/TIM-3⁺ double-positive cells indicating late T cell activation under the same co-culture conditions. In (**E**) and (**F**), data represent mean ± S.D. from three independent donors. Data in (**D**–**F**) represent mean ± SD from three independent CAR T cell or non-transduced T cell donor experiments; technical replicate wells were averaged before statistical analysis. For comparisons involving more than two conditions, one-way ANOVA followed by Dunnett’s test for multiple comparisons was used, with the untreated or corresponding control condition designated as the reference group. For two-group comparisons, a two-sided unpaired Student’s t-test was used. non-significant (ns), *p* < 0.05 (*), *p* < 0.01 (**), *p* < 0.001 (***)

From the seven human PDOs derived from PDAC patients, two PDOs, PDO1 and PDO2, showed approximately 40% and 90% CD318-positive tumor cells, respectively (Fig. 1A and S4A). In addition to the percentage of positive cells, flow-cytometric MFI measurements demonstrated heterogeneous relative CD318 expression across the PDAC PDO panel (Fig. S5). This variability provided a framework for evaluating CAR-318 T cell activity across different levels of CD318 expression. This allows to test the applicability of OrganoIDNet-derived morphological measurements under distinct expression conditions.

These CD318-positive PDOs were co-cultured either with CAR-318 T cells (presenting a transduction efficiency from 40 to 60%), or with non-transduced T cells (NTCs) from three independent donors (Fig. 1B), using the Matrigel-based “sandwich method” [7]. After 3 days of co-culture, we quantified tumor cells and CAR T cells, distinguishing early (LNGFR^+^/CD69^+^ cells) and late activation states (LNGFR^+^/TIM-3^+^ cells). From Incucyte images, OrganoIDNet was applied to determine the organoid number and area across multiple effector-to-target (E: T) ratios (Fig. 1C). CAR-318 T cells reduced the number of live tumor cells (7-AAD⁻ cells) in both PDO models in an E: T-dependent manner (Fig. 1D and S4B). At an E: T ratio of 2:1, ~ 15% killing was observed in both PDO1 and PDO2. Increasing the ratio to 5:1 resulted in ~ 30% killing in both models. At the highest ratio (8:1), CAR-318 T cells mediated ~ 45% killing in PDO1 and ~ 40% killing in PDO2. Despite differences in the proportion of CD318-positive cells and relative CD318 fluorescence intensity, comparable levels of cytotoxicity were observed across the two PDAC PDO models at the tested E: T ratios. These results indicate that CAR-318 T cells retained measurable activity across distinct CD318-expression distributions. In contrast, NTCs did not significantly affect tumor cell viability at any E: T ratio (Fig. 1D and S4B).

We next evaluated T cell dynamics by assessing CAR T cell frequency (via the expression of low-affinity nerve growth factor, LNGFR) and by evaluating their activation states. In PDO1 co-cultures, CAR T cells exhibited a 14% increase in CD69^+^ cells (Fig. 1E) and a trend toward an ~ 8% reduction in TIM-3^+^ cells (Fig. 1F), irrespective of the E: T ratio. These findings are consistent with CAR T cell activation during the assay period. Similar trends were observed in PDO2 co-cultures, with increased CD69^+^ cells (Fig. S4C) and decreased TIM-3^+^ CAR T cell numbers (Fig. S4D). To further evaluate the applicability of the assay across patient heterogeneity, we extended the analysis to four additional PDAC PDOs, PDO3, PDO5, PDO6, and PDO7 (Fig. S6). Flow-cytometric analysis demonstrated heterogeneous CD318 expression across the expanded PDO panel (Fig. S5). OrganoIDNet-based quantification of organoid number and area after co-culture revealed variable responses to CAR-318 T cells among the additional PDOs. Compared with untreated organoids and non-transduced T cell controls, CAR-318 T cells produced a marked reduction in organoid number and/or area in responsive PDO models, whereas the magnitude of the response differed between samples. These results demonstrate that the assay can capture heterogeneous CAR T cell responses across a panel of seven PDAC PDOs.

**Fig. 2:**
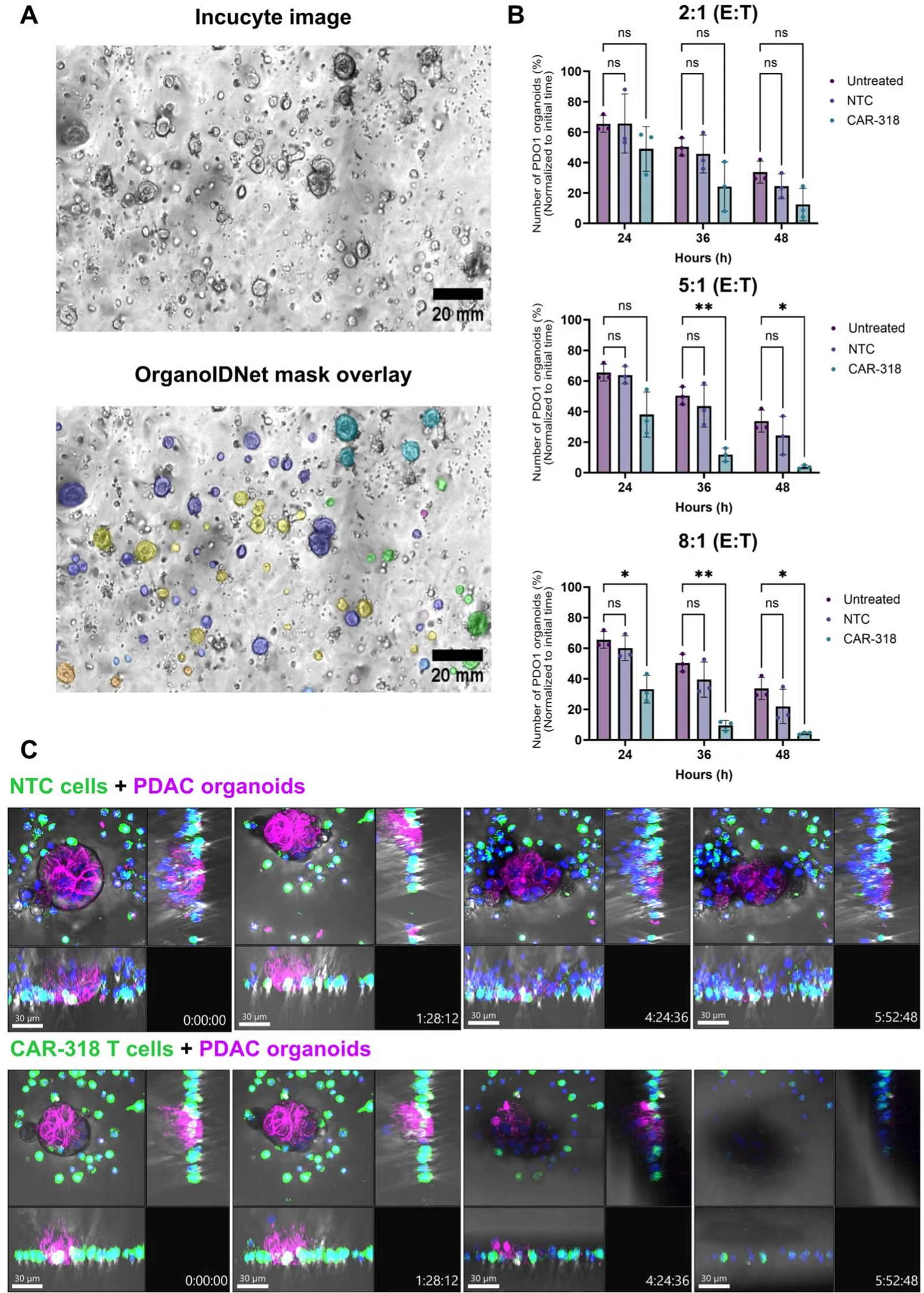
OrganoIDNet analysis of real-time human PDAC organoid responses to CAR T cell targeting. (**A**) Representative Incucyte image of PDAC PDOs co-cultured with CAR-318 T cells, illustrating OrganoIDNet-generated segmentation masks in which each organoid is assigned a distinct color. (**B**) Real-time quantification of organoid numbers across multiple time points in PDO1 PDAC organoids cultured alone (PDO1), co-cultured with CAR-318 T cells at varying effector-to-target (E: T) ratios (PDO1 + CAR-318), or co-cultured with non-transduced control T cells (PDO1 + NTC). E: T ratios correspond to CAR T cells: tumor cells (2:1, 5:1, 8:1). Data represent mean ± SD from three independent CAR T cell or non-transduced T cell donor experiments, with technical replicate wells averaged before statistical analysis. Longitudinal measurements are presented to show response kinetics. For comparisons involving more than two conditions, one-way ANOVA followed by Dunnett’s test for multiple comparisons was used, with untreated organoids as the reference group; non-significant (ns), p < 0.05 (*), p < 0.01 (**). (**C**) Representative confocal live-cell images from one of the three regions of interest (ROIs) per condition, showing PDAC organoids stained with an antibody against EpCAM (magenta) and co-cultured with either CAR-318 or NTC T cells labeled with BioTracker Cystine-FITC (green). The nuclei of the cells were stained with Hoechst 3342 (blue). Scale bars in (**C**) represent 30 μm

Using live-cell imaging in “organoid mode” on the Incucyte system, OrganoIDNet accurately segmented individual organoids, as illustrated by the mask overlays in which distinct colors represent different PDAC organoids (Fig. 2A). Consistent with our flow cytometry findings, both the number and area of PDO1 organoids were significantly reduced following exposure to CAR-318 T cells and, to a lesser extent, upon co-culture with NTCs. The most pronounced reductions occurred after 36 h, particularly in the 8:1 E: T condition, which showed ~ 40% fewer organoids (Fig. 2B) and ~ 50% reduced organoid areas (Fig. S7) compared with organoid cultures without T cells. A similar response was observed in PDO2 organoids, with substantial area reduction occurring after 48 h of co-culture, and was again most prominent in the 8:1 E: T condition, which showed ~ 50% reduction in organoid area (Fig. S7).

To directly visualize T cell-organoid interactions, we performed confocal live imaging on PDO1 organoids co-cultured with either CAR-318 T cells or NTCs (5:1 E: T ratio) using the sandwich method. As anticipated, CAR-318 T cells exhibited increased dynamic interactions and proximity to PDAC organoids. Interestingly, EpCAM-stained tumor cells were eliminated within 6 h in CAR-318 T cell co-cultures, while EpCAM expression persisted in NTC conditions (Fig. 2C).

**Fig. 3:**
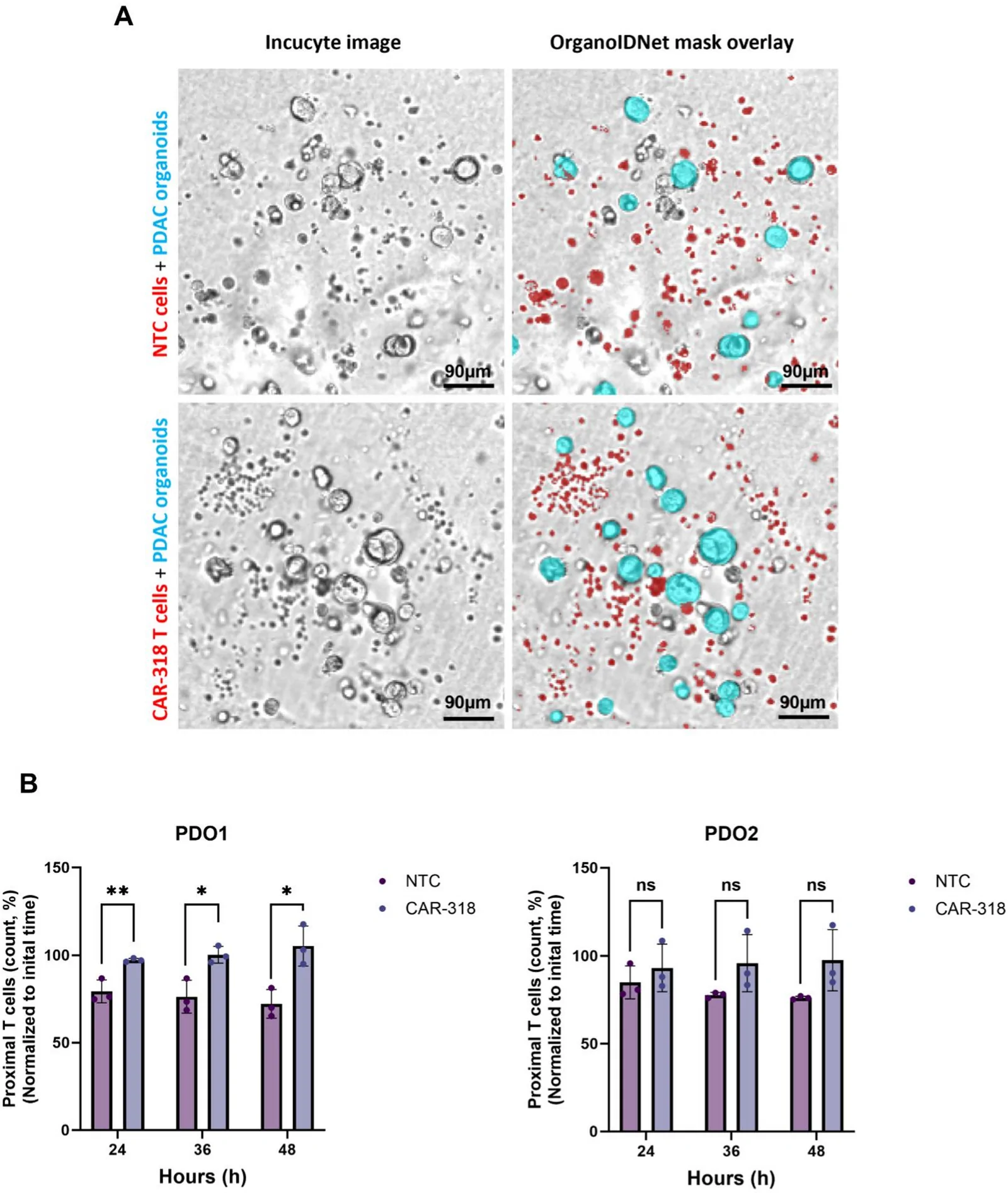
Spatial analysis from OrganoIDNet reveals close proximity of CAR-318 T cells and PDAC organoids. (**A**) Representative Incucyte image of PDAC PDOs co-cultured with NTCs or CAR-318 T cells, illustrating OrganoIDNet-generated segmentation masks in which each organoid is assigned with blue color and T cells with red color. (**B**) Real-time quantification of T cells proximity to PDO1 or PDO2 organoids across multiple time points co-cultured with NTC or co-cultured with CAR-318 T cells. Data represent mean ± SD from three independent CAR T cell or non-transduced T cell donor experiments, with technical replicate wells averaged before statistical analysis. The spatial comparisons were prespecified as CAR-318 versus non-transduced T cells within each PDO model. For direct two-group comparisons, a two-sided unpaired Student’s t-test was used; non-significant (ns), p < 0.05 (*), p < 0.01 (**). Scale bars in (**A**) represent 90 μm

To quantitatively assess the spatial interactions between CAR-CD318 T cells and PDAC organoids, OrganoIDNet was applied to segment individual organoids (blue) and define an organoid neighborhood region. T cells (red) located within this spatial zone were automatically detected and quantified from Incucyte co-culture images of organoids incubated with either CAR-CD318 T cells or non-transduced controls (NTCs) (Fig. 3A). This approach enabled measurement of T cell proximity to organoids as an indication for early infiltration and engagement. Consistent with confocal imaging observations and cytotoxicity assays, co-cultures of PDO1 and PDO2 PDAC organoids with CAR-CD318 T cells demonstrated a significantly higher density of T cells within the defined neighborhood region compared with NTCs (Fig. 3B). These results indicate enhanced spatial accumulation and interaction of CAR-CD318 T cells with PDAC organoids, supporting their targeted engagement and cytotoxic activity.

**Fig. 4:**
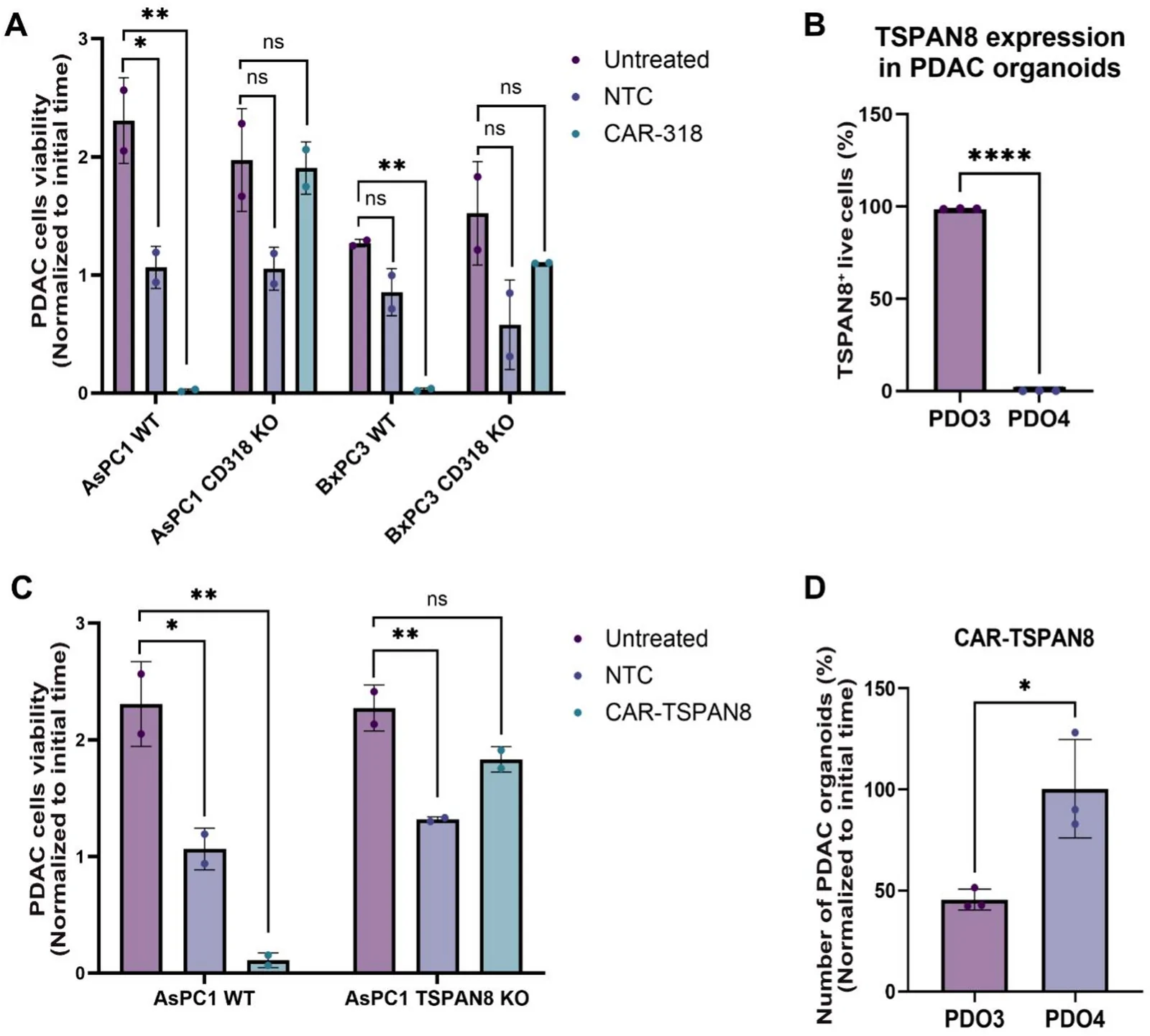
OrganoIDNet captures antigen-specific CAR T cell cytotoxicity. Cytotoxic quantification of AsPC1 and BxPC3 cell lines and their respective CD318 knockout (KO) variants after 60 h of co-culture with CAR-318 T cells. (**B**) Quantification of live TSPAN8⁺ tumor cells as a percentage of total live tumor cells in PDAC PDO3 and PDO4 organoids. (**C**) Cytotoxic quantification of AsPC1 and their corresponding TSPAN8 KO variants after 60 h of co-culture with CAR-TSPAN8 T cells. (**D**) OrganoIDNet quantification of organoid numbers after 60 h in PDO3 or PDO4 PDAC organoids co-cultured with CAR-TSPAN8 T cells. Data in (**A**) and (**C**) represent mean ± SD from two independent CAR T cell donor experiments. Data in (**B**) and (**D**) represent mean ± SD from three independent experiments; technical replicate wells were averaged before statistical analysis. For experiments involving more than two groups, one-way ANOVA followed by Dunnett’s test for multiple comparisons was used, with untreated designated as the reference group. For two-group comparisons, a two-sided unpaired Student’s t-test was used; non-significant (ns), p < 0.05 (*),p < 0.01 (**), p < 0.0001 (****)

To confirm antigen specificity of CAR-318 killing, CD318 knockout (KO) variants of AsPC1 and BxPC3 were generated (Fig. S8). CAR-318 T cells efficiently eliminated CD318-expressing wild-type (WT) cells, whereas no cytotoxicity was observed in the corresponding CD318 KO cell lines, confirming strict antigen-dependent activity (Fig. 4A).

Assessment of CD318 expression across our PDO collection revealed that all organoids expressed CD318, although at variable levels (Fig. S5). While this precluded the use of CD318-negative PDOs as specificity controls, the consistent expression across samples further supports CD318 as a broadly expressed PDAC target. To further evaluate the specificity of the OrganoIDNet analytical pipeline, we therefore used TSPAN8 as an alternative PDAC-associated antigen. TSPAN8-targeting CAR T cells (CAR-TSPAN8) have previously demonstrated potent anti-tumor activity but are limited in clinical translation due to low-level physiological expression in colon tissue [5]. Importantly, we found PDOs with high TSPAN8 levels and PDOs with non-detectable TSPAN8 expression, providing a naturally occurring antigen-negative model (Fig. 4B). CAR-TSPAN8 cells efficiently killed AsPC1 WT cells (Fig. 4C) and TSPAN8-positive PDOs (Fig. 4D), while no cytotoxicity was detected in TSPAN8 KO AsPC1 cells (Fig. 4C) or in TSPAN8-negative (Fig. 4D) organoids.

## Discussion

To overcome limitations in the in vitro evaluation of CAR T cell function, we established a stable Z-stack co-culture system combining CAR T cells with PDAC organoids. The additional application of OrganoIDNet [8], a previously established deep learning-based algorithm, to bright-field images of cancer organoids acquired using Incucyte live-cell imaging enables accurate, time-resolved quantification of cancer organoid responses to CD318-directed CAR T cell therapy. CD318-CAR T cells elicited antigen-dependent cytotoxicity across a panel of PDOs derived from seven PDAC patients with variable CD318 expression. However, the magnitude of the response varied between the different PDAC PDOs, highlighting the biological heterogeneity of patient-derived tumor models. This variability may reflect differences not only in CD318 expression, but also in the fraction of antigen-positive cells, organoid architecture, growth kinetics, and other tumor-intrinsic determinants of CAR T cell engagement and killing. Notably, the observed cytotoxicity was accompanied by increased CD69 expression and a modest reduction in TIM-3-positive CAR T cells at the endpoint. This is in line with previous in vivo studies, which demonstrated the high potency of CD318-CAR T cells for PDAC treatment [5].

Recent computational platforms have advanced organoid image analysis. OrganoID enables automated segmentation and longitudinal tracking of organoids from bright-field images, whereas Cellos, based on StarDist as well, provides high-throughput 3D segmentation and cellular-level analysis from fluorescence microscopy datasets [12, 13]. Similarly, BEHAV3D is a 3D live-cell imaging platform to characterize the behavior of engineered immune cells interacting with PDOs [14]. In this context, OrganoIDNet stands out from Cellos, OrganoID and BEHAV3D primarily because it eliminates the need for any fluorescent staining, working directly on brightfield/phase-contrast live-cell imaging overtime, whereas Cellos requires nuclear fluorescent dyes and 3D image stacks, and BEHAV3D depends on fluorescent live/dead dyes and tagged effector cells. This label-free approach avoids phototoxicity, photobleaching, and added reagent costs during longitudinal, multi-day drug-response experiments. Another advantage is that OrganoIDNet was built for organoid-immune cell co-cultures, trained directly on PDAC organoids combined with PBMCs, so it correctly segments organoids even with immune cells present in the same field of view. Neither OrganoID nor Cellos was designed or validated for this scenario, and while BEHAV3D does study immune-organoid interactions, it requires already-tracked object coordinates from software like Imaris or manual tools like Fiji/MTrackJ before it can run any analysis. Finally, OrganoIDNet delivers a whole-organoid readout directly from brightfield data, rather than reconstructing nuclei-level detail as Cellos does or relying on externally pre-processed tracking data as BEHAV3D does. Combined with publicly available pretrained, this makes OrganoIDNet a considerably more user-friendly, end-to-end pipeline than the multi-step, software-dependent workflows required by Cellos and BEHAV3D.

In summary, by integrating organoid-immune co-cultures with automated single-organoid segmentation and accurate morphological tracking, we show that OrganoIDNet transforms bright-field imaging into an automated longitudinal assay that provides multiple image-derived measurements, including organoid number, organoid area and T cell proximity. In combination with endpoint flow cytometry and confocal imaging, these measurements provide complementary temporal, morphological, and spatial information about CAR T cell-mediated PDAC organoid responses. However, the present implementation should not be considered a fully high-content assay in the broader sense, because it does not include comprehensive multiparametric fluorescence imaging or single-cell phenotypic profiling throughout the time course. Moreover, the rather short observation period of this study permits assessment of short-term killing kinetics and continued activity during the assay. However, it does not allow long-term CAR T cell durability, serial killing capacity, resistance evolution or exhaustion. By contrast, conventional assays for CAR T cell cytotoxicity, including flow cytometry-based killing assays, bioluminescence imaging, and 2D co-cultures, offer only fragmented insight into the dynamics of CAR T cell towards cancer cells. Flow cytometry remains largely restricted to end-point analysis, requires fluorescent labeling to distinguish effector and target compartments, and provides minimal information on the spatial pattern of tumor clearance. Bioluminescence-based systems permit continuous readouts but requires genetic manipulation and are influenced by metabolic state and substrate availability [12].

As we show here, 3D organoids and organoid-immune co-cultures address many of these shortcomings by more faithfully recapitulating tumor architecture, microenvironmental barriers, and patient-specific heterogeneity. So far, organoid-based assays remain technically demanding. Substantial variability in organoid size, morphology, and structural complexity across and within patient samples complicates quantitative imaging and limits assay scalability. OrganoIDNet-driven image analysis addresses some of these challenges by enabling single-organoid segmentation and objective morphological quantification, although formal scalability and reproducibility assessments remain necessary.

A useful future extension of the OrganoIDNet framework would be the integration of a dedicated deep learning-based segmentation module for T cells. Similar to the neural network currently used for organoid detection, such a model could enable automated and more accurate identification of immune cells within the imaging data. This addition would further expand the spatial analysis capabilities of OrganoIDNet, allowing a quantitative characterization of T cell-organoid interactions.

While our work advances CAR T cell live-imaging analysis, some limitations must be acknowledged. Like all organoid-based assays, our protocol is complex and technically demanding. The Matrigel-based sandwich co-culture introduces operator-dependent variability and relies on a murine-derived matrix with batch-to-batch potential differences. These differences do not fully recapitulate the dense, desmoplastic microenvironment of pancreatic tumors and may influence T cell motility and killing kinetics. Different extracellular matrices can affect immune cell behavior: for example, collagen I gels generally support amoeboid T cell migration, whereas Matrigel, a laminin- and collagen IV-rich basement membrane extract, can reduce motility and dampen activation due to its higher density and stiffness [15].

Matrigel was selected as it provides a well-standardized, basement-membrane-like environment that robustly supports 3D organoid growth, polarization, and long-term expansion. It is widely used in tumor organoid-immune co-culture models and drug-response studies [16]. To mitigate potential inhibitory effects on T cells, we reduced Matrigel to 10%, balancing organoid integrity with efficient CAR-T cell infiltration and activation, as confirmed by our data.

These challenges are partially addressed by OrganoIDNet-driven single-organoid segmentation and objective morphological quantification. Continued methodological refinement, including simplified workflows and defined matrix alternatives, will be essential to enhance reproducibility and translational relevance.

Another limitation of the present study is that the immune effector compartment consisted of CAR T cells generated from healthy donors rather than autologous T cells or tumor-infiltrating lymphocytes (TIL). Although this approach enabled comparison across three independent CAR T cell donors and provided a controlled assessment of CAR-mediated activity, it does not reproduce the patient-specific immune dysfunction, antigen experience, or exhaustion state that may be present in T cells isolated from individual patients with PDAC. Future studies should evaluate whether the imaging-derived parameters identified here are preserved when patient-matched T cells, expanded TILs, or other clinically relevant immune-cell products are paired with the corresponding PDOs.

In summary, our system combines longitudinal morphological measurements with image-based T cell proximity analysis and endpoint phenotypic characterization, providing complementary information on CAR T cell-mediated organoid responses.

## Conclusion

By combining PDAC patient-derived organoid-immune co-cultures with OrganoIDNet-based live-cell image analysis, we developed an automated longitudinal assay for quantifying short-term CAR T cell-mediated PDAC organoid responses. The assay provided continuous measurements of organoid number and area and enabled image-based assessment of T cell proximity, while endpoint flow cytometry and confocal microscopy provided complementary information on tumor-cell viability, T cell activation, and spatial association. Together, these readouts demonstrated antigen-dependent CAR T cell activity and highlighted the capacity of the platform to capture dynamic tumor-immune interactions over approximately 2 days. Further validation across a broader range of PDOs and T cell donors, including patient-matched T cells or TILs, as well as calibrated quantitative flow cytometry to determine absolute CD318 molecules per cell, serial tumor rechallenge, and longitudinal profiling of T cell phenotype and function, will help define the broader applicability of the assay. In particular, quantitative assessment of antigen density across a larger PDO panel will enable more direct investigation of the relationship between CD318 antigen abundance and CAR T cell efficacy. Such studies will further establish the scalability of the platform, its ability to capture durable treatment responses, and its potential predictive value for personalized immunotherapy.

## Supplementary Information

Below is the link to the electronic supplementary material.

Supplementary Material 1

## Data Availability

The datasets used and/or analyzed during the current study are available from the corresponding authors on reasonable request.

## Abbreviations

CAR: Chimeric antigen receptor
CD318: Cluster of differentiation 318
EpCAM: Epithelial cell adhesion molecule
E:T: Effector-to-target ratio
LNGFR: Low-affinity nerve growth factor receptor
NTC: Non-transduced T cell
PDAC: Pancreatic ductal adenocarcinoma
PDO: Patient-derived organoid
TIM-3: T cell immunoglobulin and mucin domain–containing protein 3
TIL: Tumor-infiltrating lymphocytes
7-AAD: 7-aminoactinomycin D
KO: Knockout
WT: Wild-type
