# Supplementary material for: Real-time monitoring of CAR T cell dynamics in tumor patient-derived organoids using the OrganoIDNet algorithm
Source: J Transl Med. 2026 Sep 9;24:1169. doi: 10.1186/s12967-026-08929-x (PMC13563877; doi:10.1186/s12967-026-08929-x)
Supplement: Supplementary file 1 [file 12967_2026_8929_MOESM1_ESM.docx]

Supplementary Materials for

Real-Time Monitoring of CAR T Cell Dynamics in Tumor Patient-Derived Organoids using OrganoIDNet-based algorithm

Nathalia Ferreira ^1^, Camille Dourlens ^1,2,3^, Riccardo Scodellaro ^1^, Philipp Stroebel ^4^, Daniel Schäfer ^2^, Olaf Hardt ^2^, and Frauke Alves ^1,3,5,*^

^1^Translational Molecular Imaging, Max-Planck-Institute for Multidisciplinary Sciences, 37075 Göttingen, Germany

^2^Miltenyi Biotec B.V. & Co. KG, 51429 Bergisch Gladbach, Germany

^3^Clinic of Hematology and Medical Oncology, University Medical Center Göttingen, 37075 Göttingen, Germany

^4^Institute of Pathology, University Medical Center Göttingen, 37075 Göttingen, Germany

^5^Institute for Clinical and Interventional Radiology, University Medical Center Göttingen, 37075 Göttingen, Germany

*Correspondence- [falves@gwdg.de](mailto:falves@gwdg.de) (MPINAT)

Table S1. Clinical data for the PDAC patient-derived organoid (PDO) cohort

| PDO ID ^a^ | CRU5002 TM ^b^ |
| --- | --- |
| PDO1 | 39 |
| PDO2 | 61 |
| PDO3 | 78 |
| PDO4 | 16 |
| PDO5 | 65 |
| PDO6 | 56 |
| PDO7 | 27 |

a- Patient-derived organoids identifier (ID) of each sample; b- Trial member (TM) identification according to the clinical research unit (CRU) 5002, funded by the German Research Foundation (DFG)

Fig. S2: Gating strategy for flow cytometric analysis of CD318 or TSPAN8 expression in PDAC cells or PDAC PDOs.

Gating scheme for assessing CD318 (A) or TSPAN8 (B) expression in human PDAC cells or PDOs. Cells were selected based on forward scatter area (FSC-A) versus side scatter area (SSC-A), followed by doublet discrimination using FSC-A versus FSC-H. Live cells were identified as 7-AAD⁻. Both CD318 and TSPAN8 expression were quantified within this population using CD318 versus FSC-A and TSPAN8 versus FSC-A plots, respectively.

**Fig. S3: Gating strategy for activation marker analysis of immune cells in human PDAC patient derived organoid co-cultures.**

Gating strategy for characterizing immune cell populations in human PDAC organoid-CAR T cell co-cultures. Initial gating steps excluded debris and doublets (FSC-A versus SSC-A, FSC-A versus FSC-H). Dead cell and tumor cell populations were excluded from analysis based on 7-AAD and EpCAM staining, respectively. Live CAR T cells were gated as LNGFR⁺ to distinguish CAR⁺ and CAR⁻ T cells. Early and late activation markers were assessed by the expression of CD69 and TIM-3, respectively. Activation-marker gates were established using CAR⁻ cells and the non-transduced control (NTC) and then applied to the CAR⁺ population to ensure that activation marker assessment was based on uniform, unbiased thresholds across all T cell subsets.

**Fig. S4: Flow cytometric analysis of tumor and immune cell populations in human PDAC organoids (PDO2) co-cultures with CAR-318 T cells.**

**(A)** Quantification of live CD318⁺ cells as a percentage of total live CD318⁺ tumor cells in human PDAC patient-derived organoids, PDO2. Data represent mean ± S.D. from two independent experiments. **(B)** Frequency of 7-AAD^-^ tumor cells as a percentage of total live tumor cells in PDAC PDO2 after 3-day co-culture with CAR-318 T cells (PDO2+CAR-318), non-transduced control T cells (PDO2+NTC), or in the absence of T cells (PDO2) at varying effector-to-target (E:T) ratios. Data represent mean ± S.D. from three independent CAR/NTC donor co-cultures, with technical replicate wells averaged before statistical analysis. For comparisons involving more than two conditions, one-way ANOVA followed by Dunnett's test for multiple comparisons was used, with the untreated condition; non-significant (ns), p < 0.05 (*), p < 0.01 (**). **(C)** Proportion of LNGFR⁺/CD69⁺ double-positive cells indicating early T cell activation following 3-day co-culture of CAR-318 or NTC T cells with PDAC PDO2 at different E:T ratios. **(D)** Proportion of LNGFR⁺/TIM-3⁺ double-positive cells indicating late T cell activation under the same co-culture conditions. In (C) and (D), data represent mean ± S.D. from three independent donors, with technical replicate wells averaged before statistical analysis. For direct two-group comparisons, a two-sided unpaired Student's t-test was used; non-significant (ns), p < 0.05 (*). In (B-D), E:T ratios correspond to CAR T cells: tumor cells (2:1, 5:1, 8:1).

**Fig. S5.** **CD318 expression across human PDAC PDOs.**

Quantification of CD318 expression in seven human PDAC PDOs, in comparison to the CD318-negative human breast tumor cell line MCF-7 cell line as a negative control, assessed by flow cytometry. Data are representative from two independent experiments.

**Fig. S6. OrganoIDNet analysis of CAR-318 efficacy across PDOs from 4 different PDAC patients**

Total organoid number and total organoid area were quantified after 48 h in different PDOs (PDO3, PDO5, PDO6, and PDO7 cultured alone (Untreated), co-cultured with non-transduced T cells (NTC), or co-cultured with CAR-318 T cells (CAR-318) at an E:T ratio of 5:1. Values were normalized to the corresponding initial measurement. Data represent mean ± SD from three independent experiments, with technical replicate wells averaged before statistical analysis. For comparisons involving untreated, non-transduced T-cell, and CAR-318 conditions, one-way ANOVA followed by Dunnett’s test for multiple comparisons was used with data from Untreated as prespecified controls.

Fig. S7. OrganoIDNet analysis of real-time human PDAC PDOs area in response to CAR T cell targeting.

Real-time quantification of changes in organoid area at multiple time points in PDO1 and PDO2 PDAC organoids cultured alone (Untreated), co-cultured with non-transduced control T cells (NTC), or co-cultured with CAR-318 T cells at varying effector-to-target (E:T) ratios (CAR-318). E:T ratios correspond to CAR T cells: tumor cells (2:1, 5:1, 8:1). Data represent mean ± S.D. from three independent CAR/NTC donor experiments, with technical replicate wells averaged before statistical analysis. For comparisons involving more than two conditions, one-way ANOVA followed by Dunnett's test for multiple comparisons was used, with untreated PDOs; non-significant (ns), p < 0.05 (*), p < 0.01 (**).

**Fig. S8. Evaluation of CD318 and TSPAN8 knockout PDAC cell lines.**

Successful knockout (KO) of CD318 in AsPC-1 **(A)** and BxPC-3 **(B)**, and TSPAN8 in AsPC-1 **(C)** cells, was confirmed by reduced surface expression compared with wild-type (WT) PDAC cells.
